# Associations of Plasma Fatty Acid Patterns During Pregnancy With Gestational Diabetes Mellitus

**DOI:** 10.3389/fnut.2022.836115

**Published:** 2022-05-06

**Authors:** Peiyun Li, Shan Hu, Yalun Zhu, Taoping Sun, Yue Huang, Zihui Xu, Hongjie Liu, Cheng Luo, Shiqiong Zhou, Aijun Tan, Liegang Liu

**Affiliations:** ^1^Department of Nutrition and Food Hygiene, Hubei Key Laboratory of Food Nutrition and Safety, School of Public Health, Tongji Medical College, Huazhong University of Science and Technology, Wuhan, China; ^2^The Ministry of Education Key Laboratory of Environment and Health, School of Public Health, Tongji Medical College, Huazhong University of Science and Technology, Wuhan, China; ^3^Department of Clinical Nutrition, Wuhan Children’s Hospital (Wuhan Maternal and Child Healthcare Hospital), Tongji Medical College, Huazhong University of Science and Technology, Wuhan, China; ^4^Zhuhai Center for Disease Control and Prevention, Zhuhai, China

**Keywords:** fatty acid, gestational diabetes mellitus, pattern, case-control study, pregnancy

## Abstract

**Background:**

Limited studies have explored the difference of fatty acid profile between women with and without gestational diabetes mellitus (GDM), and the results were inconsistent. Individual fatty acids tend to be interrelated because of the shared food sources and metabolic pathways. Thus, whether fatty acid patters during pregnancy were related to GDM odds needs further exploration.

**Objective:**

To identify plasma fatty acid patters during pregnancy and their associations with odds of GDM.

**Methods:**

A hospital-based case-control study including 217 GDM cases and 217 matched controls was carried out in urban Wuhan, China from August 2012 to April 2015. All the participants were enrolled at the time of GDM screening and provided fasting blood samples with informed consent. We measured plasma concentrations of fatty acids by gas chromatography–mass spectrometry, and derived potential fatty acid patterns (FAPs) through principal components analysis. Conditional logistic regression and restricted cubic spline model were used to evaluate the associations between individual fatty acids or FAPs and odds of GDM.

**Results:**

Twenty individual fatty acids with relative concentrations ≥0.05% were included in the analyses. Compared with control group, GDM group had significantly higher concentrations of total fatty acids, 24:1n-9, and relatively lower levels of 14:0, 15:0, 17:0, 18:0, 24:0, 16:1n-7, 20:1n-9,18:3n-6, 20:2n-6, 18:3n-3, 20:3n-3, 22:5n-3. Two novel patterns of fatty acids were identified to be associated with lower odds of GDM: (1) relatively higher odd-chain fatty acids, 14:0, 18:0, 18:3n-3, 20:2n-6, 20:3n-6 and lower 24:1n-9 and 18:2n-6 [adjusted odds ratio (OR) (95% confidence interval) (CI) for quartiles 4 vs. 1: 0.42 (0.23–0.76), *P*-trend = 0.002], (2) relatively higher n-3 polyunsaturated fatty acids, 24:0, 18:3n-6 and lower 16:0 and 20:4n-6 [adjusted OR (95% CI) for quartiles 4 vs. 1: 0.48 (0.26–0.90), *P*-trend = 0.018].

**Conclusion:**

Our findings suggested that two novel FAPs were inversely associated with GDM odds. The combination of circulating fatty acids could be a more significant marker of GDM development than individual fatty acids or their subgroups.

## Introduction

Gestational diabetes mellitus (GDM) refers to hyperglycemia diagnosed for the first time during pregnancy and is one of the most common complications of pregnancy in the world. According to the 10th edition of the *Diabetes Atlas* published by the *International Diabetes Federation*, an estimated 16.7% (21.1 million) of live births were affected by hyperglycemia in pregnancy in 2021. Of which, 80.3% were due to GDM (1). Although GDM usually resolves once the delivery ends, its impact on maternal and child health cannot be ignored. Women with GDM during pregnancy are at higher risk of adverse pregnancy outcomes and have an increased risk of developing GDM in subsequent pregnancies or type 2 diabetes later in life (2, 3). Babies exposed to hyperglycemia *in utero* also have a life-long higher risk of obesity and glucose intolerance (4, 5). The prevention of GDM could have far-reaching effects on the short-term and long-term health of mothers and offsprings. Hence, identifying potentially modifiable risk factors and evaluating their impact on GDM is of high priority.

Recent years, dietary fat intake has been shown to be associated with the development of insulin resistance and diabetes (6, 7). As the important composition of fat, fatty acids in tissues can reflect both the quantity and quality of dietary fat intake without being affected by recall bias and have been considered as reliable biomarkers in epidemiologic studies (8). However, up to date, limited studies have explored the difference of fatty acid profile between women with and without GDM (9). Some of them have suggested that certain fatty acids might be associated with the development of GDM, but the results were inconsistent and partly depends on the fatty acids involved (10–17). Furthermore, all the related studies only focused on traditional groups of fatty acids or a few selected individual fatty acids, whereas many circulating fatty acids of which the concentrations tend to be interrelated because of the shared food sources and metabolic pathways (18). Otherwise, fatty acid composition in previous studies were often expressed as relative amounts like mole percent or weight percent (19), which lead to the fact that a change in the level of one fatty acid might influence the amounts of others. Thus, the potential synergistic and additive effects among different fatty acids may be ignored when separately investigating the potential influence of targeted individual fatty acids or fatty acid groups on GDM risk.

To take the above complexity into account, using statistical pattern-recognition approach might be worth trying. Principal components analysis (PCA), a widely used statistical method of dimension reduction, has been applied to explore novel informative patterns such as dietary patterns or genetic patterns in complex data in the field of epidemiology (20, 21). As to biomarker panels like circulating fatty acids, PCA is also applicable and has a main effect on maximizing the information of both major and minor fatty acids. Several epidemiologic studies have identified associations of circulating or tissue fatty acid patterns (FAPs) with obesity, hypertension, cardiovascular diseases, type 2 diabetes, and cancers (22–26), which indicated that potential clinical and biological implications were existed and over that of individual fatty acids or fatty acid subgroups. However, whether FAPs during pregnancy were related to GDM odds remains unknown. We therefore conducted a matched case-control study among Chinese women to investigate the association of plasma fatty acid subgroups and individual fatty acids during pregnancy with odds of GDM initially, and further applied PCA to identify the novel FAPs associated with GDM.

## Materials and Methods

### Study Population

The hospital-based case-control study was carried out in urban Wuhan, China from August 2012 to April 2015. Pregnant women who screened for GDM at the outpatient clinics of the Department of Endocrinology, Tongji Hospital were invited to participant in the study. The inclusion criteria were as follows: (1) age ≥20 years; (2) gestational age at GDM screening ≥24 weeks; (3) singleton pregnancy. We excluded women who met any of the following items: history of diabetes (including but not limited to GDM), cardiovascular disease, cancer or other systemic diseases; pharmacologic treatment or dietary supplements use (e.g., fish oil, cod liver oil, pure docosahexaenoic acid supplements, albumen power, etc.) that might influence glucose or lipids metabolism; accompanied by other pregnancy complications; blood sample hemolysis or insufficiency; and incomplete basic information. Fasting blood samples (5 ml) were collected at the time of GDM screening using anticoagulant tubes and centrifuged at 3,000 rpm for 5 min. Plasma were separated from blood cells and stored at −80°C for further assay. The study was approved by the Ethics Committee of Tongji Medical College and was registered at www.clinicaltrials.gov as NCT05146401. All participants gave written informed consent before enrolling in the study.

### Selection of Gestational Diabetes Mellitus Cases and Controls

According to the diagnostic criteria advocated by International Association of the Diabetes and Pregnancy Study Groups (IADPSG)/WHO (27), the diagnosis of GDM can be made if one or more glucose values are above the cut points of 5.1, 10.0, and 8.5 mmol/L at fasting, 1 and 2 h during a 75-gram oral glucose tolerance test (OGTT). Controls were randomly selected and individually matched to cases by age (± 2 years), gestational age (± 2 weeks) and parity.

### Measurement of Plasma Fatty Acids

The derivatization of fatty acids in plasma was achieved by the modified direct transesterification method proposed by Lepage and Roy (28). According to the method, total plasma lipids were hydrolyzed and derived to fatty acid methyl esters (FAMEs) for further instrumental analysis. Fifty microliter of plasma was mixed with 3.6 ml of methanol-acetyl chloride (8:1; v/v) in a disposable glass tube, then 10 μl internal standard composed of non-adecanoic acid methyl ester (800 μg/ml) and 150 μl hexane were added. The sample mixture was incubated at 100°C for 1 h and cooled on ice. Afterward, 2.5 ml of 12% K_2_CO_3_ solution was slowly added to stop the reaction and neutralize the mixture. The tubes were vortexed, followed by centrifugation at 3,000 rpm for 5 min. Then, the organic layer was collected for the subsequent analysis. FAMEs were separated and analyzed by an Agilent 7890B gas chromatography (GC) coupled with an Agilent 5977A Series mass spectrometry (MS). The inlet temperature was set as 250°C. The temperature of oven was initially held at 50°C for 1 min and increased to 100°C at a rate of 25°C/min, then to 130°C at a rate of 10°C/min, and finally raised to 250°C at a rate of 5°C/min and held for 5 min. Total run time was 33 min. As to scan modes, the total ion count was used for compound identification while the selected ion monitoring was used for quantification. The dilution series of the standard mixture of 37 FAMEs and the individual standard of C22:5n-3 FAME (both from Sigma Aldrich, Poole, United Kingdom) were used for the establishment of calibration curves. The molecular weights of FAMEs, selected ions, and retention times for fatty acids detection are shown in Supplementary Table 1. The validation of analytical method was performed by reference to the European Medicines Agency Guideline on bioanalytical method validation (29). Blank samples spiked with three known concentration levels (low, medium, and high) of quality control samples were used for accuracy and precision analyses. The intra- and inter-day accuracy (expressed as recovery values) of all analytes ranged from 86.04 to 114.78% and 86.11 to 114.11%, while the intra- and inter-day precision (expressed as the coefficient of variation) of all analytes ranged from 0.63 to 7.58% and 1.15 to 12.28%, respectively. Validation results were provided in detail in the Supplementary Tables 2–4.

### Identification of Fatty Acid Patterns

In the current study, 18 trace fatty acids were excluded in the analyses because they were undetectable in most samples or the relative concentrations were <0.05% on average. After exclusion, 20 individual fatty acids, including 6 saturated fatty acids (SFAs), 4 monounsaturated fatty acids (MUFAs), and 10 polyunsaturated fatty acids (PUFAs), were used to derive FAPs through PCA. Principal components were inferred as FAPs, and we determined the number of patterns to analyze based on scree plot and eigenvalues (>1). The factor loadings reflect the contributions of individual fatty acids to principal components, which means the closer the absolute value is to 1, the greater the influence of single fatty acid on the pattern. A score of each pattern was calculated by summing fatty acid concentrations weighted by the scoring coefficients of each fatty acid.

### Covariates

Sociodemographic factors, lifestyle, and health information were collected by trained interviewers with standardized questionnaires. Height was measured using calibrated instrument. Prepregnancy body mass index (BMI) was calculated as self-reported prepregnancy weight divided by height squared (kg/m^2^). Fasting concentrations of plasma glucose (FPG), total cholesterol, triglycerides, high-density lipoprotein cholesterol (HDL-C), and low-density lipoprotein cholesterol (LDL-C) were measured using the commercial assay kits (Biosino Bio-Technology and Science, Inc.). Fasting plasma insulin (FPI) was measured by enzyme-linked immunoassay (ELISA) kits (Mercodia Company), and insulin resistance was assessed by homeostasis model assessment (HOMA) index.

### Statistical Analyses

The concentration of total fatty acids (μmol/L) was calculated as a sum of individual fatty acids (μmol/L). Individual fatty acids were expressed as mole percentage of total fatty acids. Differences of continuous variables between case and control groups were tested by the Student’s *t-*test or Mann-Whitney *U-*test, when appropriate. For categorical variables, Chi-square (χ^2^) test were adopted. Spearman partial correlation coefficients were used to estimate the relations between individual fatty acids and plasma glucose in OGTT, lipids and insulin resistance. To evaluate the associations between fatty acids and odds of GDM, we used conditional logistic regression. Odds ratios (ORs) with 95% confidence intervals (CIs) of GDM were showed in quartiles which were based on the distribution of fatty acids among controls. Tests for linear trend were conducted by treating median value for each quartile of individual fatty acids as continuous variables. False discovery rate (FDR) correction was applied in the multiple comparisons of fatty acids to reduce the false positive rate (30). Potential confounding factors including age, gestational age at blood collection, parity, prepregnancy BMI, family history of diabetes, smoking and alcohol use were adjusted in multivariable models.

To explore the association between combinations of plasma fatty acids and GDM, we did further analyses. We treated the scores of each pattern as continuous variables and evaluated the association of FAP score with GDM odds by ORs and 95% CIs through conditional logistic regression, with potential confounders adjusted. In addition, a restricted cubic spline model with four knots was used to assess the possible non-linear associations. The relationships of FAP score with indexes of glucose and lipid metabolism were also evaluated by Spearman partial correlation coefficients. Data were analyzed with SPSS 20.0 software package (SPSS, Inc.) or Stata version 13.0 (StataCorp). All *P-*values were two-sided and the threshold for statistical significance was 0.05.

## Results

### Characteristics of the Participants

Among the 683 confirmed participants who met the inclusion criteria, 15 women were excluded for previous diagnosis of diabetes, 17 women were excluded because of supplements use that might influence glucose or lipids metabolism, 3 women were excluded for incomplete basic information, 105 participants were excluded due to insufficient plasma samples (mainly consumed by previous studies). Hence, 253 women with GDM and 290 healthy pregnant women were eligible for further case-control matching by SPSS software. Finally, 217 GDM cases and 217 matched controls were selected in this study.

The demographic, anthropometric, reproductive, and metabolic characteristics of the participants were exhibited in Table 1 by case-control status. The two groups were comparable for age, gestational age, and parity. Compared to controls, GDM cases had significantly higher levels of prepregnancy BMI, FPG, 1- and 2-h post-glucose load, FPI, triglycerides and insulin resistance values, and are prone to have a family history of diabetes. The concentrations of total fatty acids were significantly higher in cases than in controls [median: 2624.96 (interquartile range 2073.66–3472.03) vs. 2458.38 (interquartile range 1857.25–2985.84) μmol/L, *P* = 0.001].

**TABLE 1:** Characteristics among women with GDM and their matched controls.

Characteristics	GDM (*N* = 217)	Non-GDM (*N* = 217)	*P*
Age (years)	30.06 ± 3.81	29.63 ± 3.76	0.239
Pre-pregnancy BMI (kg/m^2^)	22.15 ± 3.23	20.80 ± 2.64	<0.001
Parity, n (%)			1.000
1	176 (81.1)	176 (81.1)	
2	40 (18.4)	40 (18.4)	
3	1 (0.5)	1 (0.5)	
Gestational age at blood sample collection (weeks)	28.00 (26.00–30.00)	28.00 (26.00–30.00)	0.811
Family history of diabetes, n (%)	56 (25.8)	30 (13.8)	0.002
Alcohol use, n (%)	10 (4.6)	11 (5.1)	0.823
Smoking, n (%)	3 (1.4)	5 (2.3)	0.721
FPG (mmol/L)	5.21 (4.99–5.44)	4.73 (4.58–4.93)	<0.001
OGTT-1h (mmol/L)	9.78 (8.62–10.94)	7.59 (6.63–8.44)	<0.001
OGTT-2h (mmol/L)	8.65 (7.59–9.44)	6.86 (6.19–7.60)	<0.001
FPI (μU/mL)	10.38 (7.67–13.81)	8.16 (5.94–10.59)	<0.001
HOMA-IR	2.47 (1.74–3.25)	1.68 (1.25–2.31)	<0.001
Total cholesterol (mmol/L)	5.48 (4.80–6.37)	5.54 (4.78–6.14)	0.526
Triglycerides (mmol/L)	2.83 (2.26–3.73)	2.40 (1.87–3.40)	<0.001
LDL-C (mmol/L)	3.22 (2.52–4.01)	3.15 (2.44–3.85)	0.326
HDL-C (mmol/L)	1.36 (1.17–1.60)	1.38 (1.12–1.61)	0.903
Plasma total fatty acids (μmol/L)	2624.96 (2073.66–3472.03)	2458.38 (1857.25–2985.84)	0.001

*Continuous variables are shown as mean ± SDs when normally distributed, or median (IQRs) when skewed distributed. Categorical variables are shown as n (%). BMI, body mass index; FPG, fasting plasma glucose; FPI, fasting plasma insulin; GDM, gestational diabetes mellitus; HDL-C, high density lipoprotein cholesterol; HOMA-IR, homeostasis model assessment of insulin resistance; LDL-C, low density lipoprotein cholesterol; OGTT-1h, 1-h post-glucose load; OGTT-2h, 2-h post-glucose load.*

### Association of Individual Fatty Acids and Gestational Diabetes Mellitus

Plasma fatty acid composition was shown in Table 2. According to the methods, only fatty acids with relative concentrations (mol%) higher than 0.05% are displayed in the results. In both case and control groups, the most abundant fatty acids were 16:0 and 18:2n-6, whereas 24:0 and 15:0 were present in low relative concentrations. When compared GDM with control subjects, significant difference was found with several fatty acids, including five SFAs (14:0, 15:0, 17:0, 18:0, 24:0), three MUFAs (16:1n-7, 20:1n-9, 24:1n-9), and five PUFAs (18:3n-6, 20:2n-6, 18:3n-3, 20:3n-3, 22:5n-3).

**TABLE 2:** Composition (mol% of total fatty acids) of plasma fatty acids among GDM cases and non-GDM controls.

	GDM cases (*N* = 217),%	Non-GDM controls (*N* = 217),%	*P*
**SFAs**			
Total SFAs	43.77 (41.01–45.94)	43.85 (41.96–45.59)	0.547
**Even-chain SFAs**			
Total even-chain SFAs	43.45 (40.65–45.48)	43.37 (41.66–45.04)	0.635
14:0	0.34 (0.22–0.47)	0.37 (0.27–0.54)	0.006
16:0	35.47 (33.78–37.86)	35.02 (33.50–37.30)	0.154
18:0	5.86 (4.41–8.66)	7.23 (5.37–8.91)	0.001
24:0	0.07 (0.05–0.09)	0.08 (0.06–0.10)	0.001
**Odd-chain SFAs**			
Total odd-chain SFAs	0.29 (0.22–0.42)	0.35 (0.28–0.47)	<0.001
15:0	0.09 (0.07–0.11)	0.10 (0.08–0.13)	<0.001
17:0	0.21 (0.15–0.31)	0.25 (0.19–0.33)	0.001
**MUFAs**			
Total MUFAs	13.62 (11.63–15.41)	13.52 (11.99–16.14)	0.513
16:1n-7	0.50 (0.33–0.83)	0.63 (0.43–0.93)	0.003
18:1n-9	12.19 (10.00–13.89)	12.19 (10.58–14.49)	0.260
20:1n-9	0.16 (0.13–0.20)	0.19 (0.15–0.24)	<0.001
24:1n-9	0.41 (0.13–1.39)	0.24 (0.12–1.06)	0.006
**PUFAs**			
Total PUFAs	42.37 (40.12–45.59)	42.20 (39.61–44.77)	0.158
**n-6 PUFAs**			
Total n-6 PUFAs	36.15 (33.87–39.10)	35.55 (33.53–38.25)	0.187
18:2n-6	27.71 (23.68–31.47)	26.88 (23.78–30.94)	0.366
18:3n-6	0.17 (0.13–0.20)	0.19 (0.14–0.22)	0.001
20:2n-6	0.36 (0.27–0.48)	0.43 (0.33–0.56)	<0.001
20:3n-6	1.49 (1.11–2.13)	1.60 (1.114–2.20)	0.363
20:4n-6	6.02 (4.99–7.93)	6.18 (4.74–7.68)	0.688
**n-3 PUFAs**			
Total n-3 PUFAs	6.20 (5.18–7.09)	6.23 (5.11–7.09)	0.737
18:3n-3	0.45 (0.35–0.59)	0.51 (0.40–0.69)	0.001
20:3n-3	0.16 (0.11–0.20)	0.19 (0.15–0.23)	<0.001
20:5n-3	0.40 (0.31–0.51)	0.42 (0.34–0.53)	0.079
22:5n-3	0.40 (0.26–0.52)	0.43 (0.30–0.58)	0.020
22:6n-3	4.59 (3.93–5.52)	4.48 (3.70–5.24)	0.066
n-6/n-3 PUFAs	5.91 (4.91–7.13)	5.85 (4.89–7.14)	0.857

*GDM, gestational diabetes mellitus; MUFAs, monounsaturated fatty acids; PUFAs, polyunsaturated fatty acids; SFAs, saturated fatty acids.*

Spearman partial correlations between individual fatty acids and the indexes of glucose and lipid metabolism were shown in Table 3. HOMA-IR, the index that reflects insulin resistance levels, was correlated positively with 14:0, 16:0, 16:1n-7, 20:3n-6 (β = 0.14, 0.30, 0.14, and 0.15, respectively) and negatively with 15:0, 17:0, 24:0, 20:1n-9, 18:2n-6, 18:3n-6, 20:2n-6, 18:3n-3 (β =−0.13, −0.12, −0.21, −0.24, −0.21, −0.17, −0.17, and −0.12, respectively).

**TABLE 3:** Partial correlation coefficients between plasma individual fatty acids and variables of interest^a^.

	14:0	15:0	16:0	17:0	18:0	24:0	16:1	18:1	20:1	24:1	18:2n-6	18:3n-6	20:2n-6	20:3n-6	20:4n-6	18:3n-3	20:3n-3	20:5n-3	22:5n-3	22:6n-3
FPG (mmol/L)	–0.08	−0.23*	0.08	−0.18*	−0.10*	−0.14*	−0.11*	–0.01	−0.21*	0.04	–0.01	−0.15*	−0.21*	0.01	0.07	−0.13*	−0.14*	–0.06	–0.06	0.05
OGTT-1h (mmol/L)	−0.14*	−0.19*	0.01	–0.12	−0.11*	−0.14*	−0.14*	–0.03	−0.18*	0.05	0.06	−0.16*	−0.18*	–0.07	0.03	−0.16*	−0.24*	−0.15*	−0.18*	–0.01
OGTT-2h (mmol/L)	–0.02	−0.11*	0.07	–0.06	−0.12*	−0.15*	–0.02	0.03	−0.14*	0.03	–0.03	−0.12*	−0.14*	–0.01	0.03	−0.12*	−0.17*	–0.05	−0.13*	–0.01
TC (mmol/L)	−0.14*	−0.24*	0.09	−0.21*	−0.13*	−0.36*	−0.13*	0.01	−0.24*	0.06	0.06	−0.20*	−0.22*	−0.13*	–0.02	−0.19*	−0.17*	−0.19*	–0.04	0.05
TG (mmol/L)	0.17*	−0.41*	0.34*	−0.35*	−0.25*	−0.34*	0.20*	0.35*	−0.30*	–0.05	–0.09	−0.35*	−0.35*	–0.09	−0.20*	−0.11*	−0.23*	−0.30*	−0.19*	−0.26*
HDL-C (mmol/L)	–0.02	0.01	0.05	0.01	0.08	–0.03	–0.02	−0.19*	−0.10*	0.01	–0.07	–0.03	–0.03	0.09	0.17*	−0.12*	–0.01	0.07	0.19*	0.16*
LDL-C (mmol/L)	–0.09	−0.28*	0.10*	−0.28*	−0.29*	−0.12*	−0.16*	0.07	−0.23*	0.13*	0.19*	−0.14*	−0.28*	−0.20*	−0.16*	−0.20*	−0.18*	−0.25*	−0.18*	–0.08
FPI (μU/mL)	0.16*	−0.10*	0.31*	–0.11	0.02	−0.19*	0.17*	–0.01	−0.22*	–0.03	−0.21*	−0.15*	−0.15*	0.15*	0.06	−0.12*	–0.04	0.02	0.01	–0.07
HOMA-IR	0.14*	−0.13*	0.30*	−0.12*	0.03	−0.21*	0.14*	0.02	−0.24*	–0.03	−0.21*	−0.17*	−0.17*	0.15*	0.08	−0.12*	–0.07	–0.04	–0.01	–0.05

^a^Adjustment were made for age, gestational age at blood collection, parity, prepregnancy BMI, family history of diabetes, smoking and alcohol use. *P < 0.05. FPG, fasting plasma glucose; FPI, fasting plasma insulin; HDL-C, high density lipoprotein cholesterol; HOMA-IR, homeostasis model assessment of insulin resistance; LDL-C, low density lipoprotein cholesterol; OGTT-1h, 1-h post glucose load; OGTT-2h, 2-h post glucose load; TC, total cholesterol; TG, triglycerides.

In the conditional logistic regression analyses, the adjusted ORs (95% CIs) of GDM across increasing quartiles of plasma total fatty acid levels were 1.00 (referent), 1.43 (0.73–2.81), 1.11 (0.56–2.22), and 2.35 (1.24–4.47), respectively. Results for associations between fatty acid subgroups, individual fatty acids and GDM odds were displayed in Table 4 (more details could be found in Supplementary Tables 5, 6). After adjustment for age, gestational age at blood collection, parity, prepregnancy BMI, family history of diabetes, smoking, and alcohol use, total SFAs, MUFAs, or PUFAs were not significantly associated with GDM odds. However, odd-chain SFAs were inversely associated with GDM odds, whether individually or combined. Among the 20 individual fatty acids included in the analyses, 13 fatty acids were considered to be negatively associated with odds of GDM. It is worth noting that the abundance of the 13 fatty acids were all relatively low, with a proportion range of 0.08% (24:0) to 6.72% (18:0). In addition, the OR (95% CI) for GDM compared the highest with lowest quartiles of 24:1n-9 was 2.05 (1.12–3.76).

**TABLE 4:** Association between fatty acid subgroups, individual fatty acids and GDM^a^.

	Quartiles of fatty acids (%)				*P*_trend_ ^b^	*P*_FDR_ ^c^
	Q 1	Q 2	Q 3	Q 4		
SFAs	1	0.51 (0.27–0.94)	0.57 (0.31–1.04)	0.74 (0.41–1.34)	0.132	0.198
Even-chain SFAs	1	0.39 (0.20–0.75)	0.62 (0.34–1.12)	0.83 (0.45–1.53)	0.241	0.325
14:0	1	0.54 (0.29–1.01)	0.77 (0.44–1.35)	0.55 (0.31–0.99)	0.101	0.170
16:0	1	0.89 (0.46–1.74)	1.64 (0.84–3.20)	1.71 (0.85–3.45)	0.054	0.097
18:0	1	0.20 (0.09–0.44)	0.21 (0.11–0.40)	0.32 (0.16–0.63)	<0.001	<0.001
24:0	1	0.51 (0.28–0.92)	0.26 (0.12–0.54)	0.41 (0.22–0.79)	0.002	0.009
Odd-chain SFAs	1	0.33 (0.17–0.63)	0.46 (0.26–0.81)	0.45 (0.25–0.83)	0.021	0.047
15:0	1	0.85 (0.47–1.54)	0.42 (0.23–0.78)	0.48 (0.25–0.91)	0.004	0.014
17:0	1	0.31 (0.17–0.59)	0.47 (0.26–0.84)	0.43 (0.23–0.78)	0.026	0.054
MUFAs	1	0.54 (0.29–1.01)	0.96 (0.53–1.75)	0.70 (0.37–1.33)	0.475	0.513
16:1n-7	1	0.48 (0.26–0.90)	0.31 (0.16–0.61)	0.49 (0.27–0.88)	0.007	0.019
18:1n-9	1	0.61 (0.34–1.11)	0.94 (0.51–1.73)	0.65 (0.33–1.25)	0.326	0.400
20:1n-9	1	0.50 (0.26–0.96)	0.29 (0.15–0.59)	0.25 (0.12–0.52)	<0.001	<0.001
24:1n-9	1	0.95 (0.51–1.80)	0.98 (0.51–1.87)	2.05 (1.12–3.76)	0.003	0.012
PUFAs	1	1.13 (0.61–2.07)	1.07 (0.58–1.98)	1.32 (0.73–2.37)	0.381	0.429
n-6 PUFAs	1	0.86 (0.46–1.60)	0.94 (0.51–1.74)	1.43 (0.78–2.61)	0.158	0.225
18:2n-6	1	0.54 (0.29–1.03)	0.93 (0.52–1.67)	1.08 (0.60–1.97)	0.364	0.427
18:3n-6	1	0.70 (0.38–1.26)	0.34 (0.17–0.67)	0.48 (0.25–0.92)	0.008	0.020
20:2n-6	1	0.52 (0.29–0.90)	0.39 (0.21–0.73)	0.30 (0.15–0.59)	<0.001	<0.001
20:3n-6	1	0.83 (0.45–1.51)	0.64 (0.34–1.19)	0.66 (0.36–1.19)	0.123	0.195
20:4n-6	1	1.03 (0.55–1.93)	0.57 (0.30–1.09)	1.06 (0.56–1.99)	0.747	0.776
n-3 PUFAs	1	0.92 (0.51–1.67)	0.93 (0.50–1.73)	0.96 (0.53–1.72)	0.907	0.907
18:3n-3	1	0.51 (0.28–0.92)	0.54 (0.28–1.03)	0.37 (0.19–0.73)	0.007	0.019
20:3n-3	1	0.45 (0.25–0.83)	0.32 (0.17–0.64)	0.26 (0.13–0.50)	<0.001	<0.001
20:5n-3	1	0.35 (0.18–0.67)	0.49 (0.26–0.90)	0.47 (0.25–0.89)	0.030	0.058
22:5n-3	1	0.52 (0.27–0.98)	0.51 (0.27–0.98)	0.33 (0.17–0.66)	0.002	0.009
22:6n-3	1	1.01 (0.55–1.86)	0.95 (0.51–1.76)	1.34 (0.77–2.34)	0.297	0.382

*^a^Values are ORs (95% CIs). Adjustments were made for age, gestational age at blood collection, parity, prepregnancy BMI, family history of diabetes, smoking, and alcohol use.*

*^b^P_trend_ values were obtained from logistic regression by treating median value of each quartile of individual fatty acids as continuous variables.*

*^c^P_FDR_ values were P values of FDR corrections for multiple comparisons of fatty acids.*

*FDR, False discovery rate; GDM, gestational diabetes mellitus; MUFAs, monounsaturated fatty acids; PUFAs, polyunsaturated fatty acids; SFAs, saturated fatty acids.*

### Association of Novel Fatty Acid Patterns and Gestational Diabetes Mellitus

**FIGURE 1:**
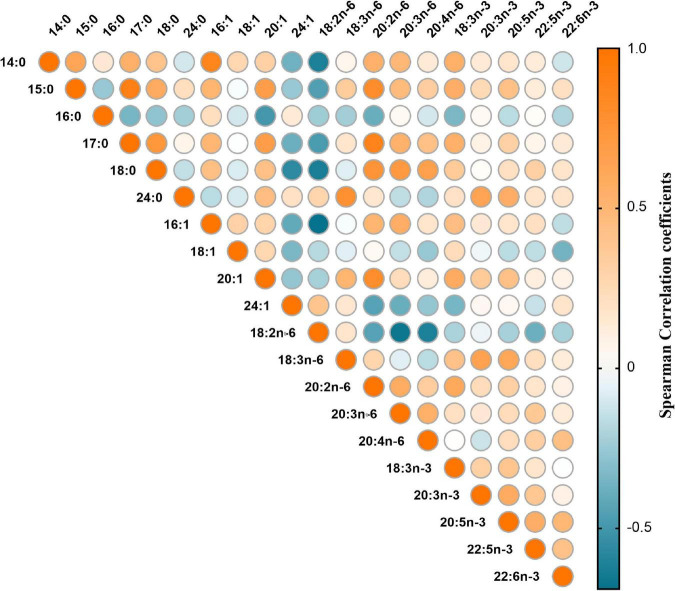
Heat map for spearman correlation matrix of 20 fatty acids measured among 217 GDM cases and matched controls.

**FIGURE 2:**
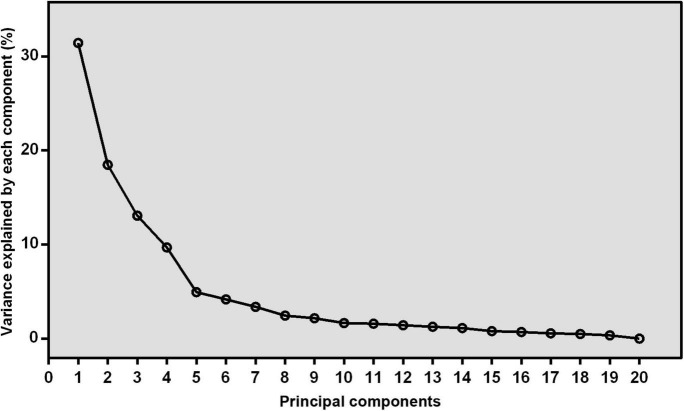
The scree plot derived from principal component analysis. The variance that explained by each component (%) was exhibited.

The correlation matrix of individual fatty acids was shown in Figure 1. Four major FAPs were identified and explained 31.41, 18.47, 13.56, and 9.68% of the overall variability, respectively. The scree plot starts to flatten from the fifth principal component (Figure 2), which was consistent with the above results.

**FIGURE 3:**
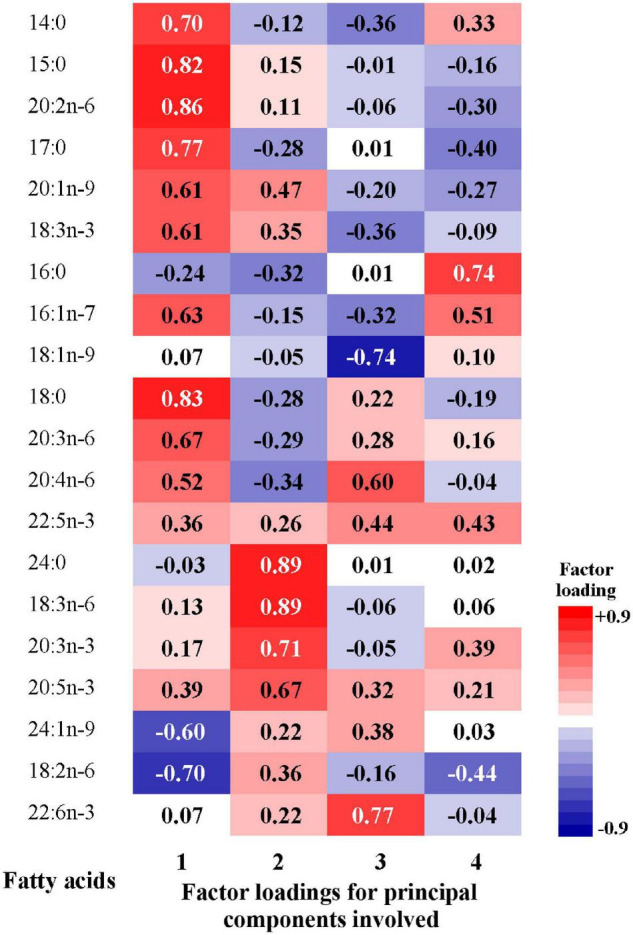
Factor loadings of the 20 fatty acids for the 1st to 4th principal components. The closer the factor loading (absolute value) is to 1, the greater the influence of single fatty acid on the pattern.

Factor loadings of the 20 fatty acids for the four patterns were shown in Figure 3. The first pattern (FAP1) was characterized by higher relative concentrations of odd-chain fatty acids, 14:0, 18:0, 18:3n-3, 20:2n-6, 20:3n-6, and lower relative concentrations of 24:1n-9 and 18:2n-6. The second pattern (FAP2) was characterized by higher abundance of n-3 PUFAs, 24:0, 18:3n-6 and lower proportions of 16:0 and 20:4n-6. The third pattern (FAP3) was featured by higher relative concentrations of 22:6n-3, 22:4n-6, 22:5n-3 and lower relative concentrations of 18:1n-9. The fourth pattern (FAP4) had moderated high factor loadings for 16:0 and 16:1n-7.

According to the scoring coefficients matrix (Supplementary Table 7), the score of each pattern can be calculated for each participant. Table 5 displays the partial correlation coefficients between FAP scores and metabolic factors. Higher FAP1 and FAP2 scores were modestly correlated with lower FPG, 1-h post glucose load, total cholesterol, triglycerides and LDL-C, and FAP2 score was further negatively correlated with 2-h post-glucose load, FPI, and HOMA-IR. Moreover, FAP3 score was positively correlated with triglycerides and HDL-C, while FAP4 was positively correlated with triglycerides, FPI, and HOMA-IR.

**TABLE 5:** Partial correlation coefficients between fatty acid pattern scores and variables of interest^a^.

	FAP1	FAP2	FAP3	FAP4
FPG (mmol/L)	−0.15*	−0.14*	0.09	0.05
OGTT-1h (mmol/L)	−0.16*	−0.16*	0.05	–0.08
OGTT-2h (mmol/L)	–0.09	−0.15*	0.02	0.02
TC (mmol/L)	−0.21*	−0.17*	0.03	0.02
TG (mmol/L)	−0.21*	−0.37*	0.32*	0.33*
HDL-C (mmol/L)	0.02	–0.02	0.25*	0.06
LDL-C (mmol/L)	−0.31*	−0.14*	−0.10*	0.04
FPI (μU/mL)	–0.01	−0.22*	0.04	0.35*
HOMA-IR	–0.03	−0.24*	0.05	0.33*

^a^Adjustments were made for age, gestational age at blood collection, parity, pre-pregnancy BMI, family history of diabetes, smoking and alcohol use. *P < 0.05. FAP, fatty acid pattern; FPG, fasting plasma glucose; FPI, fasting plasma insulin; HDL-C, high density lipoprotein cholesterol; HOMA-IR, homeostasis model assessment of insulin resistance; LDL-C, low density lipoprotein cholesterol; OGTT-1h, 1-h post glucose load; OGTT-2h, 2-h post glucose load; TC, total cholesterol; TG, triglycerides.

**FIGURE 4:**
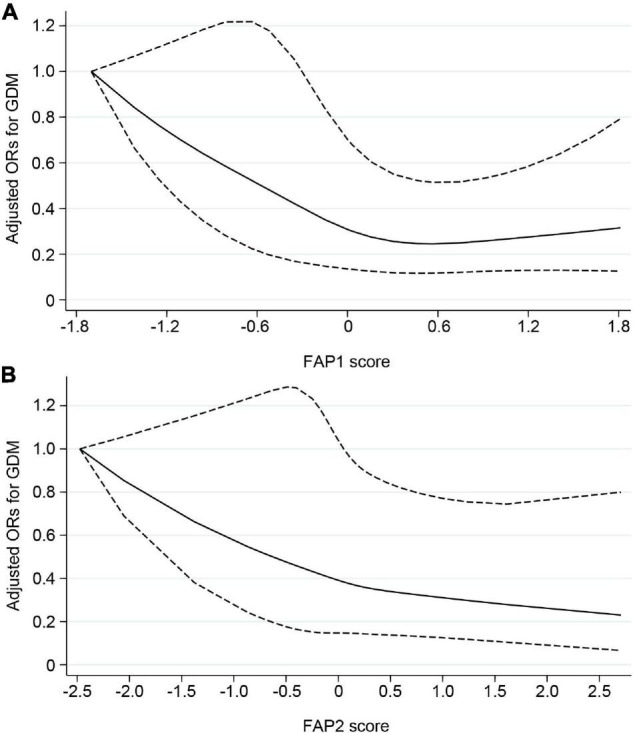
Potential non-linear associations of FAP1 **(A)** and FAP2 **(B)** with odds of GDM assessed by restricted cubic spline model. Solid line indicated adjusted ORs for GDM, and dashed line indicated 95% CIs. Adjustments were made for age, gestational age at blood collection, parity, prepregnancy BMI, family history of diabetes, smoking and alcohol use. FAP, fatty acid pattern; GDM, gestational diabetes mellitus; ORs, odds ratios; CIs, confidence intervals.

After multivariable adjustment, the FAP1 and FAP2 were inversely associated with GDM odds (Table 6). Compared with women in the lowest quartile of FAP1 score, women in the highest quartile experienced a 58% lower odds of GDM (OR, 0.42; 95% CI, 0.23–0.76). The OR (95% CI) of GDM comparing extreme quartiles of FAP2 score was 0.48 (0.26–0.90). Potential non-linear associations of FAP1 and FAP2 with odds of GDM were also found in the restricted cubic spline model (Figure 4). Nevertheless, little evidence of an association was found between quartiles of FAP3 or FAP4 scores and odds of GDM.

**TABLE 6:** ORs (95% CIs) for GDM according to quartiles of plasma fatty acid pattern scores.

	Quartiles of plasma fatty acid pattern score				*P*_trend_ ^a^
	Q 1	Q 2	Q 3	Q 4	
**FAP 1**					
N (Case/control)	91/54	50/54	34/54	42/55	
Crude model	1	0.58 (0.35–0.97)	0.40 (0.23–0.68)	0.46 (0.27–0.79)	0.001
Model 1	1	0.54 (0.31–0.95)	0.40 (0.22–0.73)	0.45 (0.26–0.81)	0.002
Model 2	1	0.50 (0.28–0.89)	0.40 (0.21–0.74)	0.42 (0.23–0.76)	0.002
**FAP 2**					
N (Case/control)	87/55	48/54	39/54	43/54	
Crude model	1	0.55 (0.33–0.93)	0.44 (0.25–0.78)	0.48 (0.28–0.82)	0.005
Model 1	1	0.47 (0.26–0.84)	0.43 (0.23–0.80)	0.50 (0.27–0.91)	0.019
Model 2	1	0.48 (0.26–0.85)	0.40 (0.20–0.77)	0.48 (0.26–0.90)	0.018
**FAP 3**					
N (Case/control)	36/54	51/55	59/54	71/54	
Crude model	1	1.42 (0.81–2.51)	1.72 (0.96–3.09)	1.98 (1.13–3.45)	0.014
Model 1	1	1.12 (0.60–2.09)	1.55 (0.81–2.98)	1.82 (0.99–3.35)	0.035
Model 2	1	1.17 (0.61–2.23)	1.48 (0.76–2.89)	1.76 (0.94–3.33)	0.058
**FAP 4**					
N (Case/control)	49/54	62/55	57/54	49/54	
Crude model	1	1.25 (0.73–2.12)	1.18 (0.69–2.00)	0.97 (0.55–1.71)	0.848
Model 1	1	1.06 (0.60–1.89)	1.02 (0.56–1.83)	1.02 (0.54–1.91)	0.996
Model 2	1	1.06 (0.58–1.96)	0.98 (0.53–1.82)	1.01 (0.52–1.95)	0.937

*^a^ P_trend_ values were obtained from logistic regression by treating median value of each quartile of FAP scores as continuous variables. FAP, fatty acid pattern; GDM, gestational diabetes mellitus.*

## Discussion

In this matched case-control study, we initially found that 13 individual fatty acids including 5 SFAs (14:0, 15:0, 17:0, 18:0, 24:0), 2 MUFAs (16:1n-7, 20:1n-9), and 6 PUFAs (18:3n-6, 20:2n-6, 18:3n-3, 20:3n-3, 20:5n-3, 22:5n-3) were negatively associated with odds of GDM, while 24:1n-9 was positively associated with GDM odds. Afterward, we identified two certain patterns of circulating fatty acids which were associated with lower odds of GDM. The first pattern was characterized by higher relative concentrations of odd-chain fatty acids, 14:0, 18:0, 18:3n-3, 20:2n-6, 20:3n-6 and lower relative concentrations of 24:1n-9 and 18:2n-6. The second pattern was characterized by higher abundance of n-3 PUFAs, 24:0, 18:3n-6, and lower proportions of 16:0 and 20:4n-6. To our knowledge, this is the first study focused on circulating FAPs related to odds of GDM. The above results suggested that the combinations of circulating fatty acids could be a more significant marker of GDM development than individual fatty acids or their subgroups.

For fatty acids with relatively high concentrations, small changes in the levels of other fatty acids may have little impact on their relative amounts or the relationships with diseases. However, for fatty acids with relatively low concentrations, the influence of others might be more considerable. In our study, individual fatty acids which were significantly associated with GDM odds were mostly of the second type. Thus, investigation of the FAPs with PCA may confer benefits over investigating individual fatty acids or fatty acid groups, and provide new insight into the joint effect of circulating fatty acids on GDM development.

The FAP1 that we identified contained positive loadings for odd-chain fatty acids, 14:0, 18:0, 18:3n-3, 20:2n-6, 20:3n-6 and negative loadings for 24:1n-9 and 18:2n-6. Although the main fatty acids involved in this pattern were distributed in various subgroups, internal correlation existed. Linoleic acid (18:2n-6), the most abundant n-6 PUFA in human circulating, is the precursor for the endogenous synthesis of other long-chain n-6 PUFAs, including 20:2n-6, 20:3n-6, and 20:4n-6 (8). A recent study conducted by Kim et al. found that low 18:2n-6 diet and estrogen significantly increased the expression of peroxisome proliferator-activated receptor α (PPAR-α), fatty acid desaturase 2 (Δ6 desaturase), and elongases of vary long chain fatty acids in rats liver (31). The fatty acid desaturases and elongases play a major role in the levels of circulating long chain PUFAs (32). Linoleic acid can compete with α-linolenic acid (18:3n-3) for rate-limiting enzyme like Δ6 desaturase and affect circulating level of 18:3n-3 and its conversion into very long chain n-3 PUFAs eventually (33). As a ligand-dependent transcription factor, PPAR-α can be activated by fatty acids and their derivatives (34), and have been found to be able to downregulate the expression of Δ9-16 and Δ9-18 desaturases which are the rate-limiting enzymes in synthesis of 16:1n-7 and 18:1n-9. The above evidence may partly explain the inverse correlation of 18:2n-6 with 18:0, 18:3n-3, 20:2n-6 and 20:3n-6 in our study. Several potential mechanisms might contribute to the protective role of FAP1 in GDM development. Firstly, 18:2n-6 is the direct precursor of hydroxyl conjugated linoleic acid. Research evidence has suggested that lowering dietary 18:2n-6 from 6.7 to 2.4% of calories for 12 weeks could markedly reduce the abundance of human plasma hydroxyl conjugated linoleic acid which is the important component of oxidized low density lipoprotein and has the effect of promoting inflammatory (35). Inflammation and dyslipidemia are considered to be the major mechanisms of insulin resistance. Secondly, 18:2n-6 competes with 18:3n-3 for conversion process. Study conducted by Ghafoorunissa et al. found that the substitution of one-third of dietary 18:2n-6 with 18:3n-3 resulted in lowered blood lipid levels and increased insulin sensitivity in sucrose fed rats, possibly due to the resulting high long chain n-3 PUFA levels in target tissues of insulin action (36). Thirdly, odd-chain fatty acids, a subclass of SFA with very low abundance, are proved to be biomarkers of dairy fat and dietary fiber intake. A large meta-analysis that pooled findings from 16 prospective cohort studies indicated that higher levels of 15:0 and 17:0 were associated with a lower risk of type 2 diabetes (37). The inverse association between odd-chain fatty acids and diabetes was considered to be partly related to dietary fiber and dairy fiber intake (38).

The FAP2 was characterized by higher abundance of n-3 PUFAs, 24:0, 18:3n-6 and lower proportions of 16:0 and 20:4n-6. As the most abundant fatty acid in circulation, 16:0 can be directly obtained from foods or synthesized from other fatty acids, amino acids or carbohydrates *via de novo* lipogenesis. The level of 16:0 in tissue was relatively stable, and changes of dietary intake seemed have little influence on its concentration because the exogenous source was counterbalanced by the endogenous biosynthesis (39). However, pathological state, chronic malnutrition, or unhealthy lifestyle factors like physical inactivity may have great impact on *de novo* lipogenesis and further affect 16:0 level (40, 41). Mounting evidence suggested that disruption in 16:0 homeostasis was associated with the development of diseases including cardiovascular diseases, metabolic diseases, neurodegenerative diseases and cancers (39). Higher tissue 16:0 could induce inflammatory responses and metabolic dysregulation which further result in dyslipidemia, hyperglycemia, and insulin resistance (42). These findings have also been validated in animal and cell researches (43, 44). In addition, an optional intake of 16:0 in a certain ratio with PUFA (including n-3 and n-6 families) was considered to have significant effect on human health, which was in line with our findings on FAP2 (40). The *in vitro* experiments showed that 22:6n-3 could reduce 16:0 induced endoplasmic reticulum stress in pancreatic β cells (45), and reverse atherosclerotic changes in human endothelial cells induced by 16:0 (46). Moreover, n-3 and n-6 PUFAs are precursors of eicosanoids which have effects in mediating inflammation and regulating immune response. Interestingly, the eicosanoids derived from n-6 PUFAs are prone to be pro-inflammatory, whereas those derived from n-3 PUFAs are anti-inflammatory (47). This suggested that n-3 PUFAs may, to some extent, improve insulin sensitivity through modulating inflammation.

Up to date, no studies have explored the potential fatty acid patters that associated with GDM odds through statistical method of dimension reduction like PCA. The results of the few studies that assessed the association of individual fatty acids with incident GDM showed some inconsistency with the present study’s findings (9). Reasons for such variability include differences in gestational age at blood sample collection, biological specimen sampled, and the method of fatty acid measurement or expression. Pooled results from two nested case-control studies conducted in China indicated that higher levels of 14:0 and 16:0 were associated with higher odds of GDM, whereas higher 18:2n-6 was associated with lower odds of GDM (14). However, the fatty acids were measured for different plasma compartments in the two studies (one study used total plasma samples and another used plasma phospholipid contents), and significant associations found in one study may not be replicated in another one. Zhang et al. found that 18:3n-3 and 22:6n-3 in early pregnancy were associated with a higher risk of GDM subtype (17). One case-control study nested in a cohort of US women suggested a beneficial role of odd-chain fatty acids, 22:5n-3 and 22:4n-6 in prevention of GDM, while 16:0, 18:3n-6, and 20:3n-6 at 10–14 weeks of pregnancy were associated with increased risk of GDM (10, 11). In normal pregnancy, plasma phospholipids in maternal circulation would increase by nearly 50% as compared to non-pregnant circulation (9), and the composition of plasma fatty acids is expected to fluctuate throughout gestation, which may lead to the discrepancy in the association between individual fatty acids and GDM risk in different trimesters on pregnancy (15, 17). Ortega-Senovilla et al. found that in the serum of women with GDM, the concentrations of most fatty acids were lower than in control women, except for 20:4n-6 and 22:6n-3, which remained the same. However, when values were expressed as a percentage of total fatty acids, different results emerged, serum from women with GDM showed significantly higher proportions of 18:2n-6, 20:4n-6, 22:6n-3, and lower proportions of 16:0, 16:1n-7, 18:1n-9, 20:5n-3 (48). The discrepancy between the above results mainly due to the difference in fatty acid expression. Only 10 individual fatty acids were measured in this study, which may lead to the possibility of lacking of comparability with other studies when fatty acids were expressed as relative concentrations. It should also be noted that the placenta plays an essential role in determining how fatty acids are transferred from maternal to embryonic circulation (49). Multiple studies have evaluated placental preference of fatty acid transport and found that the placenta places a higher preference on docosahexaenoic acid transport (9). However, GDM appears to influence the transfer of PUFAs from mother to fetus. The percentages of docosahexaenoic acid, arachidonic acid, and n-6 and n-3 PUFAs were found to be lower in the cord blood of mothers with GDM than in controls (50). Hence, the impaired transfer of some certain fatty acids through the placenta to the cord blood and fetus may be a possible factor in changing the patterns of fatty acids in GDM women.

Previous studies have explored the FAPs that related to a series of diseases such as cardiovascular disease (24), type 2 diabetes (25), metabolic syndrome (51), and cancer (52). A large nested case-cohort study indicated that a combination of fatty acids that characterized by high concentrations of 18:2n-6, odd-chain fatty acids, and very long-chain fatty acids, and low concentrations of 18:3n-6 and 16:0 was associated with lower risk of type 2 diabetes (25). Another previous study using PCA on 11 fatty acids in serum found that low 18:2n-6 factor and n-3 PUFA factor predicted metabolic syndrome development over 20 years, independent of lifestyle factors (51). Significant differences of the results on identified fatty acid patters were existed between our study and studies above, which might be attributable to several reasons. First, although metabolic syndrome, type 2 diabetes, and GDM are all metabolic diseases with insulin resistance, diverse pathogenesis, and potential risk factors still exist. Besides, circulating fatty acids could be selectively transported from pregnant women to the fetus through placenta (53), which results in the difference of fatty acid composition in the plasma of pregnant women compared with non-pregnant adults (8, 54). Furthermore, differences in study design, such as the preference of biological samples, measurement of fatty acids, sample size, and the analytical method, were all the possible reasons for the discrepancy of the results. Thus, more prospective studies are needed to validate our findings.

The major strength of our study is the application of PCA on FAPs’ exploring, making it possible to take into account the interaction of individual fatty acids. Additionally, plasma fatty acids, the objective biomarker of fatty acid status, were measured by GC–MS, which was independent of diet records and recall bias. Further, all the GDM cases included in this study were new cases and were not managed with lifestyle counseling or treated with medicine. However, several limitations should also be considered. Firstly, the sample size in the current study was relatively small. Secondly, the non-prospective design of this study disenabled us to infer the causal relations between FAPs and GDM development. The trajectory of fatty acid levels across the duration of gestation may be more informative than single-point measurement. Thus, large prospective study with longitudinal data collection is warrant in future. Thirdly, whether the FAPs identified in this study are related to certain dietary patterns or food preferences remains unclear. This could also be an important direction for future research. Fourthly, despite the adjustment for potential confounding factors in study design and statistical process, we cannot exclude the possibility that residual confounders existed.

In conclusion, in this matched case-control study, we identified two novel FAPs that were inversely associated with GDM odds. The combination of circulating fatty acids could be a more significant marker of GDM development than individual fatty acids or their subgroups. Prospective studies in other populations are needed to validate our findings, and explore how to optimize FAPs during pregnancy to achieve better health outcomes.

## Data Availability Statement

The raw data supporting the conclusions of this article will be made available by the authors, without undue reservation.

## Ethics Statement

The studies involving human participants were reviewed and approved by the Ethics Committee of Tongji Medical College. The patients/participants provided their written informed consent to participate in this study.

## Author Contributions

PL, AT, and LL designed the research. PL, SH, YZ, TS, YH, ZX, HL, and SZ contributed to the data collection and analysis. PL wrote the manuscript. CL, AT, and LL edited the manuscript. LL and AT are the guarantors of this work and have full access to all the data in the study and take responsibility for the integrity of the data and the accuracy of the data analysis. All authors read and approved the final manuscript.

## Conflict of Interest

The authors declare that the research was conducted in the absence of any commercial or financial relationships that could be construed as a potential conflict of interest. The reviewer XL declared a shared affiliation with the authors PL, SH, YZ, TS, YH, ZX, HL, CL, SZ, and LL to the handling editor at the time of review.

## Publisher’s Note

All claims expressed in this article are solely those of the authors and do not necessarily represent those of their affiliated organizations, or those of the publisher, the editors and the reviewers. Any product that may be evaluated in this article, or claim that may be made by its manufacturer, is not guaranteed or endorsed by the publisher.

## Abbreviations

BMI: body mass index
FAME: fatty acid methyl ester
FAP: fatty acid pattern
FPG: fasting plasma glucose
FPI: fasting plasma insulin
GC–MS: gas chromatography–mass spectrometry
GDM: gestational diabetes mellitus
HDL-C: high density lipoprotein cholesterol
HOMA-IR: homeostasis model assessment of insulin resistance
LDL-C: low density lipoprotein cholesterol
MUFA: monounsaturated fatty acid
OGTT: oral glucose tolerance test
PCA: principal components analysis
PUFA: polyunsaturated fatty acid
SFA: saturated fatty acid.
