# Supplementary material for: Associations of Plasma Fatty Acid Patterns During Pregnancy With Gestational Diabetes Mellitus
Source: Front Nutr. 2022 May 6;9:836115. doi: 10.3389/fnut.2022.836115 (PMC9121815; doi:10.3389/fnut.2022.836115)
Supplement: Supplementary file 1 [file Table_1.pdf]

SUPPLEMENTARY DATA

Supplementary Table 1. GC-MS information on fatty acids detection

Fatty acids	Corresponding FAMEs	Molecular weight of FAMEs	Selected ions (m/z)	Retention time (min)
6:0	Methyl hexanoate	130.18	74, 87	6.487
8:0	Methyl octanoate	158.24	74, 87	8.454
10:0	Methyl decanoate	186.29	74, 87	11.162
11:0	Methyl undecanoate	200.32	74, 87	12.687
12:0	Methyl laurate	214.34	74, 87	14.316
13:0	Methyl tridecanoate	228.37	74, 87	15.943
14:0	Methyl myristate	242.40	74, 87	17.610
14:1n-5	Methyl myristoleate	240.38	55, 74	18.278
15:0	Methyl pentadecanoate	256.42	74, 87	19.212
15:1n-5	Methyl cis-10-pentadecenoate	254.41	55, 69	19.904
16:0	Methyl palmitate	270.45	74, 87	20.833
16:1n-7	Methyl palmitoleate	268.43	55, 69	21.304
17:0	Methyl heptadecanoate	284.48	74, 87	22.314
17:1n-7	cis-10-Heptadecanoic acid methyl ester	282.46	55, 69	22.830
18:0	Methyl stearate	298.50	74, 87	23.843
18:1n-9t	trans-9-Elaidic acid methyl ester	296.49	55, 69	24.057
18:1n-9	cis-9-Oleic acid methyl ester	296.49	55, 69	24.228
18:2n-6t	Methyl linolelaidate	294.47	67, 81	24.544
18:2n-6	Methyl linoleate	294.47	67, 81	24.944
19:0	Methyl nonadecanoate	312.53	74, 87	25.217
18:3n-6	Methyl γ-linolenate	292.45	79, 67	25.379
18:3n-3	Methyl linolenate	292.45	79, 67	25.838
20:0	Methyl arachidate	326.56	74, 87	26.618
20:1n-9	Methyl cis-11-eicosenoate	324.54	55, 69	26.991
20:2n-6	cis-11,14-Eicosadienoic acid methyl ester	322.52	81, 67	27.707
21:0	Methyl heneicosanoate	340.58	74, 87	27.897
20:3n-6	cis-8,11,14-Eicosatrienoic acid methyl ester	320.51	79, 67	28.127
20:4n-6	cis-5,8,11,14-Eicosatetraenoic acid methyl ester	318.49	79, 91	28.394
20:3n-3	cis-11,14,17-Eicosatrienoic acid methyl ester	320.51	79, 95	28.559
22:0	Methyl behenate	354.61	74, 87	29.182
20:5n-3	cis-5,8,11,14,17-Eicosapentaenoic acid methyl ester	316.48	79, 91	29.248
22:1n-9	Methyl erucate	352.59	55, 69	29.562

22:2n-6	cis-13,16-Docosadienoic acid methyl ester	350.58	81, 67	30.274
23:0	Methyl tricosanoate	368.63	74, 87	30.384
24:0	Methyl lignocerate	382.66	74, 87	31.747
22:5n-3	Methy all-cis-7,10,13,16,19-docosapentaenoate	344.53	79, 91	32.042
24:1n-9	Methyl nervonate	380.65	55, 69	32.230
22:6n-3	cis-4,7,10,13,16,19-Docosahexaenoic acid methyl ester	342.51	79, 91	32.348

GC-MS, gas chromatography - mass spectrometry; FAMEs, fatty acid methyl esters.

Supplementary Table 2. Lower limit of quantification and the calibration curve range of individual fatty acids

Analytes	Lower limit of quantification [a] (μg/mL)	Calibration curve range [b] (μg/mL)	Linearity r^2 values
6:0	0.02	0.10-10	0.9984
8:0	0.02	0.10-10	0.9986
10:0	0.02	0.10-10	0.9987
11:0	0.02	0.05-10	0.9994
12:0	0.02	0.10-10	0.9994
13:0	0.02	0.05-10	0.9999
14:0	0.02	0.10-20	0.9998
14:1n-5	0.05	0.10-10	0.9999
15:0	0.02	0.05-25	0.9999
15:1n-5	0.05	0.10-10	0.9999
16:0	0.02	0.15-150	0.9997
16:1n-7	0.06	0.10-50	0.9999
17:0	0.04	0.05-25	0.9999
17:1n-7	0.06	0.10-10	0.9999
18:0	0.02	0.10-100	0.9999
18:1n-9t	0.06	0.10-10	0.9994
18:1n-9	0.06	0.10-100	0.9995
18:2n-6t	0.06	0.10-10	0.9996
18:2n-6	0.06	0.10-100	0.9999
18:3n-6	0.06	0.10-25	0.9999
18:3n-3	0.05	0.10-50	0.9999
20:0	0.02	0.10-10	0.9997
20:1n-9	0.06	0.10-25	0.9999
20:2n-6	0.06	0.10-50	0.9999
21:0	0.01	0.05-10	0.9999
20:3n-6	0.06	0.10-50	0.9999
20:4n-6	0.06	0.10-50	0.9999
20:3n-3	0.05	0.10-10	0.9999
22:0	0.02	0.10-10	0.9990
20:5n-3	0.06	0.10-50	0.9999
22:1n-9	0.06	0.10-10	0.9999

22:2n-6	0.05	0.10-10	0.9999
23:0	0.02	0.05-10	0.9999
24:0	0.02	0.10-10	0.9998
22:5n-3	0.10	0.50-60	0.9994
24:1n-9	0.06	0.10-50	0.9990
22:6n-3	0.06	0.10-50	0.9999

[a] The lower limit of quantification of analytes was calculated on the basis of a signal-to-noise ratio of 10:1.

[b] The calibration curve range of analytes was determined by the lower limit of quantification and the actual concentration range of plasma fatty acids in adults acquired from pre-experiment and previous studies.

Supplementary Table 3. Accuracy for the determination of individual fatty acids [a].

Analyte	Intra-day accuracy (n = 6) (%)			Inter-day accuracy (n = 6) (%)		
	Low QC level	Medium QC level	High QC level	Low QC level	Medium QC level	High QC level
6:0	113.91	92.81	106.73	111.42	108.21	103.96
8:0	114.78	106.86	110.90	108.71	102.53	104.40
10:0	112.05	87.08	98.09	106.79	96.89	101.60
11:0	98.89	106.54	95.60	96.82	102.85	98.70
12:0	97.26	105.80	92.70	102.78	99.77	96.00
13:0	95.35	104.40	95.77	95.30	99.41	95.38
14:0	93.84	97.61	96.31	109.26	91.33	93.90
14:1n-5	96.43	103.70	97.72	96.88	97.46	95.42
15:0	91.47	102.31	97.99	101.32	101.85	94.03
15:1n-5	99.56	91.70	96.81	95.78	86.11	92.97
16:0	95.02	87.10	94.43	101.68	108.55	111.63
16:1n-7	95.07	97.66	97.64	94.66	104.42	97.24
17:0	94.32	95.31	101.68	104.60	113.44	103.90
17:1n-7	98.74	96.84	97.39	91.68	95.03	93.70
18:0	96.61	92.87	97.67	106.52	112.94	109.48
18:1n-9t	88.74	97.10	91.98	92.40	90.81	94.78
18:1n-9	86.04	86.52	89.78	88.75	95.53	105.85
18:2n-6t	96.45	92.36	91.10	89.84	94.27	97.63
18:2n-6	92.71	90.63	89.71	98.74	110.04	112.26
18:3n-6	98.70	106.90	99.08	102.60	105.89	93.56
18:3n-3	94.65	95.10	97.95	90.47	103.88	95.39
20:0	98.64	102.68	96.09	96.92	99.06	93.34
20:1n-9	112.30	108.51	106.21	96.87	113.09	97.61
20:2n-6	95.79	94.53	97.81	109.24	103.88	92.81
21:0	102.32	98.65	99.77	108.98	97.24	92.69
20:3n-6	90.22	91.70	96.55	109.98	113.67	107.27
20:4n-6	96.24	94.53	94.28	113.93	110.38	96.98
20:3n-3	95.06	107.89	99.63	89.27	104.14	93.54
22:0	92.03	98.63	97.42	90.24	90.53	91.25
20:5n-3	95.46	94.74	94.37	111.21	98.39	95.66
22:1n-9	86.20	104.77	98.63	114.11	98.36	93.26

22:2n-6	112.42	98.41	97.67	104.86	97.41	96.98
23:0	98.83	101.55	103.37	98.52	96.15	97.69
24:0	103.10	92.66	103.50	103.99	94.27	93.22
22:5n-3	92.03	93.57	98.40	101.08	96.54	93.42
24:1n-9	93.59	95.54	100.73	86.99	98.15	90.82
22:6n-3	87.35	97.91	97.89	93.83	113.90	110.90

[a] Accuracy was expressed as recovery values and determined for low, medium and high QC levels. For an analyte, the low QC level was set as the lower limit of calibration curve range, the medium QC level was set as 50% of the upper limit of calibration curve range, and the high QC level was set as 100% of the upper limit of calibration curve range.

QC, quality control.

Supplementary Table 4. Precision for the determination of individual fatty acids [a].

Analyte	Intra-day precision (n = 6) (%)			Inter-day precision (n = 6) (%)		
	Low QC level	Medium QC level	High QC level	Low QC level	Medium QC level	High QC level
6:0	7.28	6.45	3.05	12.28	10.42	10.72
8:0	2.66	3.53	7.58	5.46	5.50	5.91
10:0	1.53	2.91	2.97	6.19	2.70	4.32
11:0	1.10	1.46	3.48	5.11	3.25	3.88
12:0	1.05	1.11	3.75	3.68	3.77	3.51
13:0	1.15	1.24	4.00	3.15	4.41	3.79
14:0	3.90	1.47	4.52	6.24	2.90	4.16
14:1n-5	6.60	1.46	4.41	3.24	4.42	3.55
15:0	2.11	1.41	4.17	2.32	4.00	3.89
15:1n-5	3.43	2.13	5.15	2.08	3.17	3.57
16:0	2.11	3.18	5.24	5.60	3.61	3.85
16:1n-7	3.66	1.86	4.21	3.54	5.21	4.09
17:0	1.39	3.53	4.19	4.01	3.44	4.28
17:1n-7	2.93	1.36	3.24	3.86	4.00	3.53
18:0	2.95	5.03	4.84	2.47	4.77	4.05
18:1n-9t	2.76	5.26	5.15	5.60	4.95	6.77
18:1n-9	4.68	5.63	6.37	3.41	4.60	4.18
18:2n-6t	2.65	3.23	3.14	4.01	3.46	4.61
18:2n-6	2.99	6.77	4.71	3.56	7.31	4.22
18:3n-6	2.77	1.90	4.09	6.35	4.18	4.49
18:3n-3	6.04	2.49	5.84	3.78	6.01	5.21
20:0	1.06	0.96	3.46	4.46	4.51	3.16
20:1n-9	1.28	1.87	3.65	5.97	1.94	3.99
20:2n-6	1.40	2.73	4.84	1.15	3.60	4.15
21:0	5.48	2.46	5.55	4.76	7.42	3.76
20:3n-6	1.35	5.71	4.12	5.09	5.82	3.81
20:4n-6	0.80	6.55	3.89	2.47	4.40	3.25
20:3n-3	1.46	1.01	3.59	7.67	6.05	5.12
22:0	2.32	1.10	3.63	7.63	6.59	3.45
20:5n-3	1.93	3.56	4.95	6.76	2.76	4.04
22:1n-9	6.40	1.20	4.01	8.61	5.23	3.56

22:2n-6	2.84	3.40	4.40	6.33	4.84	3.02
23:0	2.41	0.63	4.29	2.54	4.22	2.97
24:0	3.54	3.11	4.09	5.34	7.64	4.14
22:5n-3	2.78	2.54	2.83	2.99	4.39	4.91
24:1n-9	2.42	2.07	5.89	1.90	5.23	4.85
22:6n-3	1.06	5.48	2.62	4.49	5.31	2.59

[a] Precision was expressed as the coefficient of variation and determined for low, medium and high QC levels. For an analyte, the low QC level was set as the lower limit of calibration curve range, the medium QC level was set as 50% of the upper limit of calibration curve range, and the high QC level was set as 100% of the upper limit of calibration curve range.

QC, quality control.

Supplementary Table 5. ORs (95% CIs) for GDM according to quartiles of plasma fatty acid groups [a].

	Quartiles of fatty acid groups (%)				P_{trend} [b]
	Q 1	Q 2	Q 3	Q 4	
SFAs	≤41.96	41.97-43.85	43.86-45.59	>45.59	
N (Case/control)	75/54	37/54	42/55	63/54	
Crude model	1	0.52 (0.30-0.90)	0.59 (0.35-1.01)	0.85 (0.50-1.45)	0.243
Model 1	1	0.50 (0.27-0.90)	0.59 (0.33-1.06)	0.79 (0.44-1.43)	0.173
Model 2	1	0.51 (0.27-0.94)	0.57 (0.31-1.04)	0.74 (0.41-1.34)	0.132
Even-chain SFAs	≤41.66	41.66-43.37	43.38-45.04	>45.04	
N (Case/control)	76/55	29/54	47/54	65/54	
Crude model	1	0.40 (0.22-0.72)	0.68 (0.40-1.16)	0.90 (0.53-1.54)	0.418
Model 1	1	0.39 (0.21-0.73)	0.63 (0.35-1.13)	0.90 (0.49-1.63)	0.315
Model 2	1	0.39 (0.20-0.75)	0.62 (0.34-1.12)	0.83 (0.45-1.53)	0.241
Odd-chain SFAs	≤0.28	0.29-0.35	0.36-0.47	>0.47	
N (Case/control)	103/55	27/53	40/54	47/55	
Crude model	1	0.29 (0.16-0.52)	0.43 (0.26-0.72)	0.46 (0.27-0.78)	0.012
Model 1	1	0.33 (0.18-0.61)	0.46 (0.27-0.81)	0.49 (0.27-0.87)	0.029
Model 2	1	0.33 (0.17-0.63)	0.46 (0.26-0.81)	0.45 (0.25-0.83)	0.021
MUFAs	≤12.00	12.01-13.52	13.53-16.15	>16.15	
N (Case/control)	65/54	39/54	71/54	42/55	
Crude model	1	0.62 (0.36-1.08)	1.14 (0.67-1.93)	0.65 (0.38-1.13)	0.279
Model 1	1	0.47 (0.26-0.88)	0.89 (0.49-1.61)	0.65 (0.35-1.19)	0.298
Model 2	1	0.54 (0.29-1.01)	0.96 (0.53-1.75)	0.70 (0.37-1.33)	0.475
PUFAs	≤39.61	39.62-42.20	42.21-44.77	>44.77	
N (Case/control)	45/54	57/54	50/54	65/55	
Crude model	1	1.23 (0.72-2.10)	1.07 (0.62-1.84)	1.38 (0.82-2.32)	0.262
Model 1	1	1.16 (0.64-2.11)	1.06 (0.59-1.91)	1.38 (0.78-2.43)	0.293
Model 2	1	1.13 (0.61-2.07)	1.07 (0.58-1.98)	1.32 (0.73-2.37)	0.381
n-6 PUFAs	≤33.53	33.54-35.55	35.56-38.25	>38.25	
N (Case/control)	48/55	45/53	53/54	71/55	
Crude model	1	0.99 (0.57-1.71)	1.11 (0.66-1.88)	1.47 (0.87-2.49)	0.102
Model 1	1	0.88 (0.48-1.62)	1.05 (0.58-1.90)	1.46 (0.82-2.62)	0.111
Model 2	1	0.86 (0.46-1.60)	0.94 (0.51-1.74)	1.43 (0.78-2.61)	0.158
n-3 PUFAs	≤5.11	5.12-6.23	6.24-7.09	>7.09	
N (Case/control)	50/55	60/53	53/55	54/54	
Crude model	1	1.23 (0.73-2.08)	1.06 (0.62-1.84)	1.09 (0.65-1.82)	0.899

Model 1	1	1.02 (0.57-1.82)	1.07 (0.59-1.95)	1.02 (0.58-1.79)	0.921
Model 2	1	0.92 (0.51-1.67)	0.93 (0.50-1.73)	0.96 (0.53-1.72)	0.907
n-6/n-3	≤4.893	4.894-5.850	5.851-7.135	>7.135	
N (Case/control)	54/54	52/55	57/54	54/54	
Crude model	1	0.94 (0.54-1.65)	1.05 (0.62-1.78)	1.00 (0.59-1.71)	0.907
Model 1	1	0.80 (0.42-1.49)	0.82 (0.45-1.49)	1.04 (0.57-1.88)	0.792
Model 2	1	0.68 (0.36-1.30)	0.79 (0.43-1.45)	1.08 (0.58-1.99)	0.654

[a] Values are ORs (95% CIs). Model 1 adjusted for age and pre-pregnancy BMI. Model 2 adjusted for Model 1 plus gestational age at blood collection, parity, family history of diabetes, smoking and alcohol use (Yes vs. No).

[b] P_{trend} values were obtained from logistic regression by treating median value of each quartile of fatty acid groups as continuous variables.

Supplementary Table 6. ORs (95% CIs) for GDM according to quartiles of plasma individual fatty acids [a].

	Quartiles of fatty acids (%)				P_{trend} [b]
	Q 1	Q 2	Q 3	Q 4	
14:0	≤0.27	0.28-0.37	0.38-0.54	>0.54	
N (Case/control)	83/55	36/53	61/54	37/55	
Crude model	1	0.45 (0.26-0.79)	0.79 (0.48-1.28)	0.46 (0.27-0.79)	0.020
Model 1	1	0.55 (0.30-1.01)	0.82 (0.48-1.40)	0.57 (0.32-1.01)	0.119
Model 2	1	0.54 (0.29-1.01)	0.77 (0.44-1.35)	0.55 (0.31-0.99)	0.101
15:0	≤0.08	0.09-0.10	0.11-0.13	>0.13	
N (Case/control)	86/55	61/53	36/55	34/54	
Crude model	1	0.76 (0.46-1.28)	0.44 (0.26-0.76)	0.42 (0.24-0.73)	<0.001
Model 1	1	0.84 (0.48-1.47)	0.45 (0.25-0.81)	0.48 (0.27-0.92)	0.005
Model 2	1	0.85 (0.47-1.54)	0.42 (0.23-0.78)	0.48 (0.25-0.91)	0.004
16:0	≤33.50	33.51-35.02	35.03-37.30	>37.30	
N (Case/control)	48/55	41/54	66/54	62/54	
Crude model	1	0.91 (0.51-1.63)	1.51 (0.84-2.72)	1.44 (0.79-2.62)	0.124
Model 1	1	0.87 (0.45-1.67)	1.52 (0.79-2.89)	1.70 (0.86-3.33)	0.051
Model 2	1	0.89 (0.46-1.74)	1.64 (0.84-3.20)	1.71 (0.85-3.45)	0.054
17:0	≤0.19	0.20-0.25	0.26-0.33	>0.33	
N (Case/control)	98/54	32/55	39/53	48/55	
Crude model	1	0.34 (0.20-0.60)	0.44 (0.27-0.74)	0.48 (0.29-0.81)	0.024
Model 1	1	0.33 (0.18-0.60)	0.47 (0.27-0.83)	0.47 (0.26-0.84)	0.037
Model 2	1	0.31 (0.17-0.59)	0.47 (0.26-0.84)	0.43 (0.23-0.78)	0.026
18:0	≤5.37	5.38-7.23	7.24-8.91	>8.91	
N (Case/control)	102/54	36/55	32/53	47/55	
Crude model	1	0.29 (0.16-0.53)	0.30 (0.17-0.54)	0.41 (0.23-0.71)	0.001
Model 1	1	0.23 (0.11-0.47)	0.23 (0.12-0.43)	0.36 (0.19-0.69)	<0.001
Model 2	1	0.20 (0.09-0.44)	0.21 (0.11-0.40)	0.32 (0.16-0.63)	<0.001
24:0	≤0.07	0.07-0.08	0.09-0.10	>0.10	
N (Case/control)	90/55	57/54	29/53	41/55	
Crude model	1	0.63 (0.38-1.05)	0.30 (0.16-0.57)	0.45 (0.25-0.79)	0.001
Model 1	1	0.52 (0.29-0.92)	0.29 (0.15-0.57)	0.44 (0.24-0.83)	0.002
Model 2	1	0.51 (0.28-0.92)	0.26 (0.12-0.54)	0.41 (0.22-0.79)	0.002
16:1n-7	≤0.43	0.44-0.63	0.64-0.93	>0.93	
N (Case/control)	89/54	49/54	40/54	39/55	
Crude model	1	0.55 (0.32-0.94)	0.46 (0.26-0.79)	0.48 (0.28-0.80)	0.004

Model 1	1	0.58 (0.32-1.06)	0.36 (0.19-0.68)	0.50 (0.28-0.89)	0.007
Model 2	1	0.48 (0.26-0.90)	0.31 (0.16-0.61)	0.49 (0.27-0.88)	0.007
18:1n-9	≤10.58	10.59-12.19	12.20-14.49	>14.49	
N (Case/control)	66/55	43/53	65/55	43/54	
Crude model	1	0.71 (0.42-1.80)	1.02 (0.59-1.74)	0.66 (0.38-1.15)	0.250
Model 1	1	0.57 (0.32-1.02)	0.87 (0.48-1.59)	0.61 (0.32-1.15)	0.206
Model 2	1	0.61 (0.34-1.11)	0.94 (0.51-1.73)	0.65 (0.33-1.25)	0.326
20:1n-9	≤0.15	0.16-0.19	0.20-0.24	>0.24	
N (Case/control)	92/54	60/54	35/54	30/55	
Crude model	1	0.58 (0.33-1.01)	0.32 (0.17-0.59)	0.27 (0.14-0.50)	<0.001
Model 1	1	0.52 (0.27-0.97)	0.29 (0.15-0.58)	0.24 (0.12-0.49)	<0.001
Model 2	1	0.50 (0.26-0.96)	0.29 (0.15-0.59)	0.25 (0.12-0.52)	<0.001
24:1n-9	≤0.12	0.13-0.24	0.25-1.06	>1.06	
N (Case/control)	50/55	42/54	38/53	87/55	
Crude model	1	0.87 (0.49-1.53)	0.81 (0.46-1.42)	1.76 (1.04-2.97)	0.003
Model 1	1	0.94 (0.50-1.75)	0.90 (0.48-1.68)	1.92 (1.07-3.46)	0.004
Model 2	1	0.95 (0.51-1.80)	0.98 (0.51-1.87)	2.05 (1.12-3.76)	0.003
18:2n-6	≤23.78	23.79-26.88	26.89-30.94	>30.94	
N (Case/control)	56/55	38/54	62/54	61/54	
Crude model	1	0.70 (0.41-1.22)	1.11 (0.67-1.84)	1.08 (0.64-1.83)	0.408
Model 1	1	0.56 (0.30-1.03)	0.95 (0.54-1.68)	1.07 (0.60-1.92)	0.379
Model 2	1	0.54 (0.29-1.03)	0.93 (0.52-1.67)	1.08 (0.60-1.97)	0.364
18:3n-6	≤0.14	0.15-0.19	0.20-0.22	>0.22	
N (Case/control)	72/55	68/53	38/55	39/54	
Crude model	1	0.97 (0.58-1.61)	0.52 (0.30-0.91)	0.56 (0.32-0.97)	0.011
Model 1	1	0.71 (0.40-1.26)	0.37 (0.19-0.69)	0.52 (0.28-0.95)	0.010
Model 2	1	0.70 (0.38-1.26)	0.34 (0.17-0.67)	0.48 (0.25-0.92)	0.008
20:2n-6	≤0.33	0.34-0.43	0.44-0.56	>0.57	
N (Case/control)	94/55	50/54	43/54	30/54	
Crude model	1	0.55 (0.33-0.92)	0.45 (0.26-0.79)	0.33 (0.19-0.58)	<0.001
Model 1	1	0.52 (0.30-0.91)	0.42 (0.23-0.77)	0.32 (0.17-0.60)	<0.001
Model 2	1	0.52 (0.29-0.90)	0.39 (0.21-0.73)	0.30 (0.15-0.59)	<0.001
20:3n-6	≤1.14	1.15-1.60	1.61-2.20	>2.20	
N (Case/control)	62/55	58/53	48/55	49/54	
Crude model	1	0.97 (0.57-1.64)	0.78 (0.46-1.34)	0.82 (0.49-1.38)	0.355
Model 1	1	0.87 (0.49-1.56)	0.69 (0.38-1.25)	0.69 (0.39-1.23)	0.158
Model 2	1	0.83 (0.45-1.51)	0.64 (0.34-1.19)	0.66 (0.36-1.19)	0.123

20:4n-6	≤4.74	4.75-6.18	6.19-7.68	>7.68	
N (Case/control)	51/55	65/53	40/54	61/55	
Crude model	1	1.36 (0.79-2.35)	0.81 (0.46-1.43)	1.25 (0.72-2.18)	0.852
Model 1	1	1.10 (0.60-2.03)	0.60 (0.32-1.13)	1.15 (0.62-2.13)	0.949
Model 2	1	1.03 (0.55-1.93)	0.57 (0.30-1.09)	1.06 (0.56-1.99)	0.747
18:3n-3	≤0.40	0.41-0.51	0.52-0.69	>0.69	
N (Case/control)	88/55	50/54	47/53	32/55	
Crude model	1	0.57 (0.34-0.95)	0.51 (0.29-0.89)	0.34 (0.19-0.61)	<0.001
Model 1	1	0.52 (0.29-0.93)	0.53 (0.29-0.98)	0.38 (0.20-0.73)	0.006
Model 2	1	0.51 (0.28-0.92)	0.54 (0.28-1.03)	0.37 (0.19-0.73)	0.007
20:3n-3	≤0.15	0.16-0.19	0.20-0.23	>0.23	
N (Case/control)	91/54	52/54	44/55	30/54	
Crude model	1	0.56 (0.33-0.95)	0.44 (0.25-0.78)	0.35 (0.20-0.60)	<0.001
Model 1	1	0.44 (0.24-0.80)	0.33 (0.18-0.64)	0.27 (0.14-0.52)	<0.001
Model 2	1	0.45 (0.25-0.83)	0.32 (0.17-0.64)	0.26 (0.13-0.50)	<0.001
20:5n-3	≤0.34	0.35-0.42	0.43-0.53	>0.53	
N (Case/control)	83/54	35/54	54/54	45/55	
Crude model	1	0.43 (0.25-0.75)	0.66 (0.39-1.12)	0.54 (0.32-0.92)	0.047
Model 1	1	0.37 (0.20-0.69)	0.54 (0.30-0.97)	0.51 (0.28-0.94)	0.046
Model 2	1	0.35 (0.18-0.67)	0.49 (0.26-0.90)	0.47 (0.25-0.89)	0.030
22:5n-3	≤0.30	0.31-0.43	0.44-0.58	>0.58	
N (Case/control)	73/55	51/53	54/54	39/55	
Crude model	1	0.71 (0.42-1.21)	0.71 (0.41-1.22)	0.49 (0.28-0.88)	0.022
Model 1	1	0.52 (0.28-0.97)	0.55 (0.29-1.02)	0.36 (0.19-0.71)	0.005
Model 2	1	0.52 (0.27-0.98)	0.51 (0.27-0.98)	0.33 (0.17-0.66)	0.002
22:6n-3	≤3.70	3.71-4.48	4.49-5.24	>5.24	
N (Case/control)	46/55	53/54	51/53	67/55	
Crude model	1	1.14 (0.67-1.95)	1.11 (0.64-1.93)	1.37 (0.84-2.24)	0.212
Model 1	1	1.06 (0.58-1.93)	1.08 (0.59-1.97)	1.38 (0.80-2.38)	0.229
Model 2	1	1.01 (0.55-1.86)	0.95 (0.51-1.76)	1.34 (0.77-2.34)	0.297

[a] Values are ORs (95% CIs). Model 1 adjusted for age and pre-pregnancy BMI. Model 2 adjusted for Model 1 plus gestational age at blood collection, parity, family history of diabetes, smoking and alcohol use (Yes vs. No).

[b] P_{trend} values were obtained from logistic regression by treating median value of each quartile of individual fatty acids as continuous variables.

Supplementary Table 7. Coefficients to calculate the scores of fatty acid patterns [a]

Individual FAs	Scoring coefficients			
	FAP 1	FAP 2	FAP 3	FAP 4
14:0	0.111	-0.032	-0.142	0.171
15:0	0.131	0.041	-0.004	-0.081
16:0	-0.038	-0.086	-0.001	0.383
17:0	0.123	-0.074	0.004	-0.207
18:0	0.132	-0.075	0.087	-0.097
24:0	-0.005	0.241	0.005	0.010
16:1n-7	0.100	-0.040	-0.128	0.266
18:1n-9	0.011	-0.015	-0.295	0.051
20:1n-9	0.098	0.126	-0.080	-0.137
24:1n-9	-0.094	0.058	0.151	0.014
18:2n-6	-0.111	0.097	-0.062	-0.228
18:3n-6	0.020	0.242	-0.025	0.030
20:2n-6	0.137	0.029	-0.024	-0.153
20:3n-6	0.107	-0.079	0.113	0.083
20:4n-6	0.083	-0.092	0.239	-0.022
18:3n-3	0.098	0.096	-0.142	-0.048
20:3n-3	0.027	0.192	-0.019	0.204
20:5n-3	0.062	0.181	0.126	0.109
22:5n-3	0.058	0.070	0.177	0.221
22:6n-3	0.012	0.060	0.308	-0.023

[a] The score of each pattern is calculated by summing the fatty acid levels weighted by the scoring coefficients.

Abbreviation: FAP, fatty acid pattern.
